# A Case of Rapidly Deteriorating Lymphangitic Carcinomatosis in a Patient with Stage IV Pancreatic Cancer

**DOI:** 10.7759/cureus.4421

**Published:** 2019-04-09

**Authors:** Hafiz M Aslam, Cassandra Zhi, Muhammad Nadeem, Mohammad Arsalan, Sara L Wallach

**Affiliations:** 1 Internal Medicine, Seton Hall University / Hackensack Meridian School of Medicine, Trenton, USA; 2 Internal Medicine, Drexel University College of Medicine, Philadelphia, USA

**Keywords:** pancreatic cancer, pulmonary lymphangitic carcinomatosis (plc), lymphangitic carcinomatosis

## Abstract

Pulmonary lymphangitic carcinomatosis (PLC) is defined as a tumor in the lung lymphatic vessels. It is rarely seen as a result of malignancy and generally carries a poor prognosis. Proper knowledge of the clinical presentation is important for physicians to be aware of in order to consider a diagnosis of PLC. We present the case of a 52-year-old Caucasian gentleman who initially came to the hospital with a three-week history of dyspnea. In the hospital, a diagnosis of Stage 4 pancreatic cancer with a lymphangitic spread of the tumor to the lungs was made. Despite optimal treatment, the patient required mechanical ventilation for acute hypoxic respiratory failure.

PLC is a relatively rare manifestation of malignancy; yet, it generally carries a poor prognosis. It may occur during the course of malignancy or may represent as the first findings in malignancy. It is important for physicians to be aware of the clinical presentations of PLC in order to ensure timely treatment. Oftentimes, PLC can be diagnosed through clinical judgment alone without the need for radiological support and other invasive measures.

## Introduction

Pulmonary lymphangitic carcinomatosis (PLC) is defined as a tumor in the lung lymphatic vessels. Tumor cells can invade the interstitium resulting in thickened interstitial septa and bronchovascular bundles. Due to its rare presentation and diagnostic delay, PLC is oftentimes diagnosed by autopsy as opposed to biopsy. It is a rare manifestation of malignancy and generally carries a poor prognosis [1-5]. The true incidence of lymphangitic carcinomatosis is not definitively known since most of the diagnoses are made post-mortem.

The risk of lymphangitic carcinomatosis is high with adenocarcinoma of the uterus, breast, lung, colon, cervix, melanoma, pancreas, and stomach. Many of these malignancies involve thoracic lymph nodes. This has led to a theory that these tumors can cause lymphangitis via a retrograde flow to the lungs, possibly through transdiaphragmatic passage (peritoneal carcinomatosis) [1, 6]. Another study theorizes that the tumor can spread to the lungs hematogenously and then, once inside the lungs, spreads through the lymphatics [7].

Presenting symptoms of PLC are similar to those of interstitial lung disease and include dyspnea, cough, hemoptysis, fatigue, weight loss, and fever. In this report, we will present a case secondary to pancreatic cancer, a combination that has been relatively rare in the written literature.

## Case presentation

A 52-year-old Caucasian gentleman presented to the hospital with a three-week history of shortness of breath. His past medical history was notable for diabetes mellitus type 2, human immunodeficiency virus (HIV) controlled with highly active retroviral medications, and chronic hepatitis C. His shortness of breath was mainly exertional and was associated with a dry cough. He denied fever, hemoptysis, wheezing, and chest pain. He reported a 60-pound weight loss in the few months prior to this illness. He was a seven-year former smoker with a pack/year index of 40. He denied alcohol and recreational drug use. His family history was notable for coronary artery disease in both parents. His medications included metformin and a combination pill of elvitegravir, cobicistat, emtricitabine, and tenofovir. His cluster of differentiation-4 (CD4) count was checked within the past year and was found to be 364. He reported compliance with all medications.

He was seen in an urgent care clinic and was prescribed levofloxacin with little improvement in his symptoms. He was referred to the hospital after computed tomography (CT) of the chest showed diffuse infiltrates. The chest x-ray was not available for review at the time of his hospital visit. 

**Figure 1:**
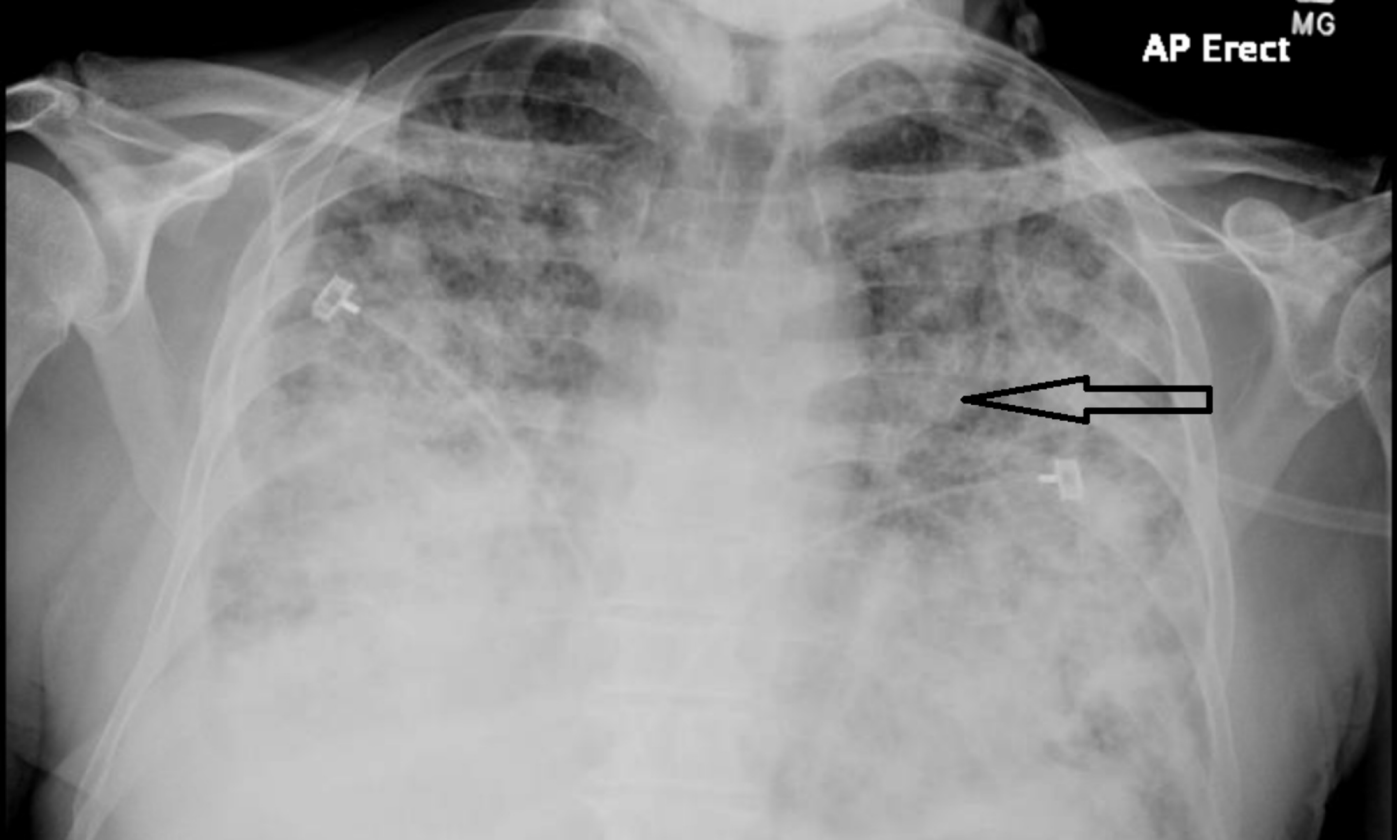
Diffuse, bilateral reticulonodular opacification with Kerley B lines is noted.

**Figure 2:**
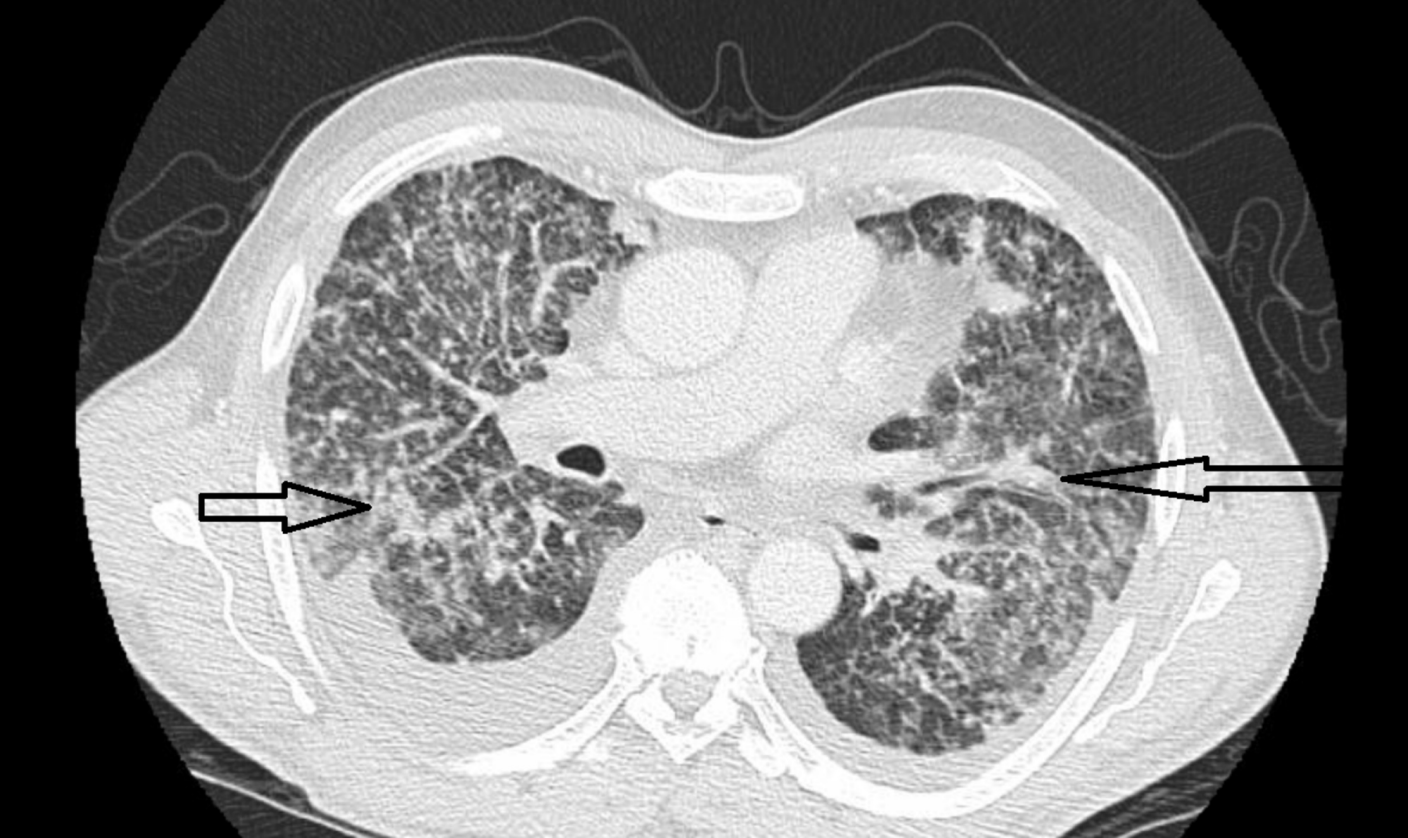
Peribronchovascular and interlobular septal thickening Irregular, nodular or smooth, peribronchovascular fissure and interlobular septal thickening. Thickening of fissure as a result of the involvement of the lymphatics concentrated in the subpleural interstitium.

His chest x-ray and chest CT showed diffuse reticulonodular opacities involving all lung lobes, as well as bilateral pleural effusions (Figures 1-2). Given his history of HIV, empiric treatment for pneumocystis pneumonia with sulfamethoxazole/trimethoprim was started. A CT scan of the abdomen and pelvis, which was done to evaluate for potential malignancy given his history of significant weight loss, revealed a mass in the body of the pancreas, as well as a left paraaortic mass.

The origin of the paraaortic mass was uncertain on the CT scan but could be from the left adrenal gland as opposed to paraaortic lymphadenopathy.

Diagnostic thoracentesis was done, and the cytology showed clusters of gland-forming cells consistent with a diagnosis of adenocarcinoma. A diagnosis of Stage 4 pancreatic cancer with the lymphangitic spread of the tumor to the lungs was made. The repeat CD4 count was 365. Respiratory cultures for bacteria and fungi were negative. Antibiotic therapy was stopped. A pancreatic biopsy was planned, but the patient’s respiratory status declined before the biopsy could be done. He was intubated and mechanically ventilated for acute hypoxic respiratory failure. The family decided to withdraw mechanical support after one week and the patient succumbed to death. The total time between the patient’s first medical contact and his death was less than six weeks.

Interstitial pulmonary diseases and infectious diseases were considered as the preliminary diagnoses in our case presentation because of the patient’s clinical picture, smoking, and HIV history. Differential diagnoses of sarcoidosis, interstitial lung disease (ILD), pneumocystis pneumonia, vasculitis, pneumonia, lymphoma, and Kaposi’s sarcoma should also be considered.

## Discussion

Pulmonary lymphangitic carcinomatosis was first described by Andral in uterine cancer in 1948 [3]. It is most commonly secondary to malignancies of the uterus, breast, lung, colon, cervix, melanoma, pancreas, or stomach [7-9]. PLC may occur during the course of the disease or it may represent the very first finding, as seen in this case. It is rarely reported as the first finding in pancreatic cancer. In most cases, pulmonary symptoms mask the findings of the primary neoplasm [7, 10]. Thus, the initial presentation is usually dyspnea and a longstanding dry cough [2].

The lymphatics in the lungs are seen in a peribronchovascular, centrilobular, interlobular, and subpleural distribution. Access to the lymphatic vessels is due to direct invasion of nearby vessels (e.g., thoracic lymphatics). It is presumed that tumor cells trapped within the lymphatics result in local obstruction and fluid accumulation. Bronchovascular bundle and alveolar septal thickening may be due to local edema, and the presence of nodules in some cases may suggest that tumor cells in lymphangitic carcinomatosis can grow locally [6-7].

CT findings of pulmonary lymphangitic carcinomatosis are divided on the basis of involvement of the interlobular septa (peripheral interstitial thickening) and bronchoalveolar bundles (axial interstitial thickening). Patients with axial involvement have been reported to have longer mean survival times than those with peripheral involvement [11]. The most common reported cause of death is respiratory failure. Bronchovascular bundle and interlobular septal thickening, ground-glass opacity, pleural effusion, mediastinal lymphadenopathy, and nodular lesions are the usual radiological findings [12].

On the basis of chest x-rays and modes of evolution, PLC has been divided into four categories [9]. Type I shows progressive diffuse linear shadows in both lung fields without hilar enlargement (e.g., stomach cancer). Type II shows progressive radiating linear shadows from bilateral enlarged hilar lymph nodes. Type III shows a pattern of lymphatic spread from one portion of the lung to another, which was proven to be a hilar-type bronchogenic carcinoma. Type IV, which differs from Type III by a mass shadow in the lung parenchyma, was proven to be the lymphatic spread of parenchymal-type bronchogenic carcinoma.

Various clinical studies and case series have found that the most common histological pattern of PLC was adenocarcinoma [9, 13]. Histopathologic evaluation is confirmatory, but the diagnosis of PLC is often made on the basis of clinical and radiological findings because of the rapid deterioration of the patient’s condition. Studies have reported that clinical and radiological findings are accurate in the diagnosis of PLC in 92% of cases with diffuse interstitial pulmonary disease [6, 12].

Even with early diagnosis, the prognosis of PLC is poor with approximately 50% of patients dying within three months of their first respiratory symptoms [7]. However, in a case report by Kikuchi et al., combination treatment with cisplatin was shown to have promising treatment results [14].

## Conclusions

In conclusion, in the presence of a chronic cough, along with dyspnea and a clinical picture suggestive of ILD with a significant weight loss, a diagnosis of PLC should be considered. Diagnosis of PLD can be made through clinical judgment with radiological support and invasive measures are often not necessary.
